# COVID-19 vaccination and BA.1 breakthrough infection induce neutralising antibodies which are less efficient against BA.4 and BA.5 Omicron variants, Israel, March to June 2022 

**DOI:** 10.2807/1560-7917.ES.2022.27.30.2200559

**Published:** 2022-07-28

**Authors:** Limor Kliker, Neta Zuckerman, Nofar Atari, Noam Barda, Mayan Gilboa, Ital Nemet, Bayan Abd Elkader, Ilana S Fratty, Hanaa Jaber, Ella Mendelson, Sharon Alroy-Preis, Yitshak Kreiss, Gili Regev-Yochay, Michal Mandelboim

**Affiliations:** 1 Central Virology Laboratory, Public Health Services, Ministry of Health and Sheba Medical Center, Tel-Hashomer, Israel; 2 Sackler Faculty of Medicine, Tel-Aviv University, Tel-Aviv, Israel; 3 Sheba Medical Center, Tel-Hashomer, Israel; 4 Israel Center for Disease Control, Israel Ministry of Health, Sheba Medical Center, Tel-Hashomer, Ramat-Gan, Israel; 5 Public Health Services, Ministry of Health, Jerusalem, Israel

**Keywords:** COVID-19, BNT162b2 vaccination, Neutralizing, Omicron, BA.4/5

## Abstract

This work evaluated neutralising antibody titres against wild type (WT) SARS-CoV-2 and four Omicron variants (BA.1, BA.2, BA.4 and BA.5) in healthcare workers who had breakthrough BA.1 infection. Omicron breakthrough infection in individuals vaccinated three or four times before infection resulted in increased neutralising antibodies against the WT virus. The fourth vaccine dose did not further improve the neutralising efficiency over the third dose against all Omicron variants, especially BA.4 and BA.5. An Omicron-specific vaccine may be indicated.

Several severe acute respiratory syndrome coronavirus 2 (SARS-CoV-2) variants of concern (VOC) have emerged during the coronavirus disease (COVID-19) pandemic [1]. Omicron is now, in July 2022, the dominant virus around the globe [2] and has four main sub-variants: BA.1, BA.2, BA.4, and BA.5. These variants contain more than 30 mutations in their spike protein compare with the Alpha and Delta variants [3,4]. Although they cause milder disease than previously reported VOC [5], they are highly transmissible, the existing vaccines Comirnaty (BNT162b2 mRNA, Pfizer/BioNTech) and Spikevax (mRNA-1273, Moderna) are less effective [6] and the Omicron variants have decreased susceptibility to therapeutic monoclonal antibodies [7]. When compared with BA.2, the BA.4 and BA.5 sub-variants carry three additional mutations in their spike protein, which have raised concerns regarding their potential to evade neutralising antibodies, thereby further compromising the effectiveness of COVID-19 vaccines and therapeutic monoclonal antibodies [8].

There is still limited information about whether individuals immunised with COVID-19 vaccine are protected against Omicron BA.4 and BA.5 compared with other strains. Here, we describe the appearance of the Omicron variants in Israel in March to June 2022 and the neutralising antibody response against these variants.

## The Omicron variants of concern in Israel

In order to identify SARS-CoV-2 variants circulating in the country, a national surveillance consortium for SARS-CoV-2 sequencing was established in Israel in December 2020. 

**Figure 1:**
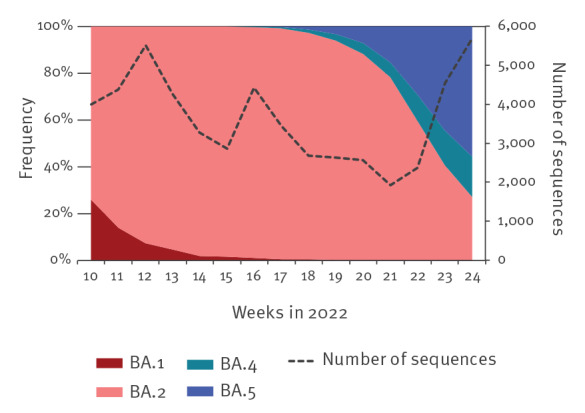
SARS-CoV-2 variants, Israel, March–June 2022 (n = 49,810)

The frequency of SARS-CoV-2 variants BA.1, BA.2, BA.4 and BA.5 in Israel was monitored by whole genome sequencing of SARS-CoV-2-positive samples (Figure 1). Between March 2022 and June 2022, a total of 49,810 sequences were analysed. Throughout most of this period, BA.2 was the dominant variant. The BA.4 and BA.5 variants were first detected in mid-April 2022 (week 15, Figure 1), whereas BA.5 became more prevalent than BA.4 at the beginning of June 2022 (week 21, Figure 1).

SARS-CoV-2: severe acute respiratory syndrome coronavirus 2.

Frequency of SARS-CoV-2 variants as detected by whole genome sequencing of SARS-CoV-2-positive samples.

## Neutralisation efficiency against SARS-CoV-2 Omicron variants

We evaluated neutralising antibody titres against wild type (WT) sub-lineage B.1.1.50 and the four Omicron variants (BA.1, BA.2, BA.4 and BA.5) in 41 healthcare workers (HCW) who had BA.1 infection and were evaluated within 30 days following positive PCR result. None of these HCW had been infected with other SARS-CoV-2 variants. We obtained sera from three HCW cohorts. The first cohort included in the analysis were 11 HCW who had never been vaccinated (Cohort 1 in the Table). Sera were obtained from this group 1 month following BA.1 infection. The second cohort included 15 HCW who had been vaccinated with the Comirnaty vaccine three times (Cohort 2 in the Table). Sera were obtained from this cohort 5 months after the third dose. Cohort 2 was then vaccinated with the fourth dose and sera were obtained 1 month later. The HCW in Cohort 2 had a breakthrough infection with Omicron BA.1 and sera were obtained 1 month later. Hence, a total of 45 serum samples were taken from Cohort 2 at various time points. The third cohort included 15 HCW who were vaccinated three times with the Comirnaty vaccine and had a breakthrough infection with BA.1; sera were obtained from them 1 month after the infection (Cohort 3 in the Table). We compared the geometric mean titres (GMTs) in the different cohorts, using the unvaccinated individuals as the baseline comparison. Demographic data and vaccination information of all HCW participating in this study is provided in the Table. 

**Table:** Details of study participants, healthcare workers recovered from SARS-CoV-2 BA.1 breakthrough infection, Israel, March–June 2022 (n = 41)

	Cohort 1	Cohort 2	Cohort 3
Number of participants	11	15	15
Median age in years (range)	41 (31–58)	57 (33–73)	48.5 (28–68)
Average age	41.9	55	48.14
Male	3	7	4
Female	8	8	11
Average days post third vaccination (range)	NA	142.46 (121–166)	134.84 (117–154)
Average days post fourth vaccination (range)	NA	15.86 (12–21)	NA
Average days post positive PCR result (range)	30.72 (3–32)	16.06 (12–26)	15.3 (10–20)

NA: not applicable; SARS-CoV-2: severe acute respiratory syndrome coronavirus 2.

Cultured virus samples were obtained from SARS-CoV-2-infected individuals and subjected to whole genome sequencing (Illumina COVID-seq kit on Illumina NovaSeq, Cambridge, United Kingdom) to identify the WT (hCoV19/Israel/CVL-45526-ngs/2020), Omicron BA.1 (hCoV-19/Israel/CVL-n49814/2021), Omicron BA.2 (hCoV-19/Israel/CVL-n51046/2022), Omicron BA.4 (hCoV-19/Israel/CVL-n51653/2022) and Omicron BA.5 (hCoV-19/Israel/CVL-n51658/2022). To test the neutralisation capacity of the sera, a median tissue culture infectious dose (100 TCID50) of WT and Omicron (BA.1, BA.2, BA.4 and BA.5) SARS-CoV-2 isolates were incubated with serially diluted (1:8 to 1:16,384) inactivated serum (30 min, 56 °C) in 96-well plates for 60 min at 33 °C. Virus–serum mixtures were then added to VERO-E6 cells and incubated for 5 days at 33 °C, after which cells were stained with Gentian violet dye (1%). Neutralising capacity was determined by the highest serum dilution at which no cytopathic effect was observed. The GMT and its 95% confidence interval (CI) are reported for each variant by cohort.

**Figure 2:**
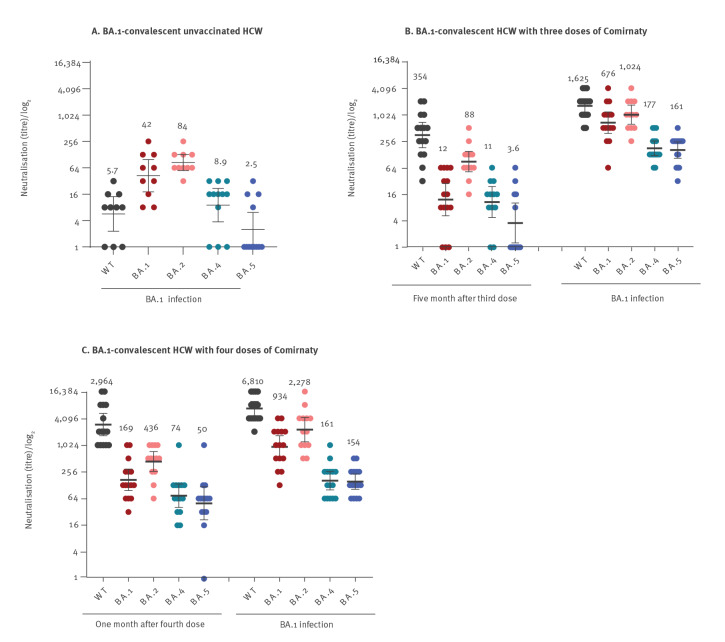
Microneutralisation of different SARS-CoV-2 variants, sera from healthcare workers in relation to vaccination and BA.1 infection, Israel, March–June 2022 (n = 41)

Omicron BA.1 infection in unvaccinated HCW induced lower neutralisation efficiency (compared with neutralisation observed following vaccination) against BA.1 and BA.2, but practically no effective neutralisation against BA.4, BA.5 or WT (Figure 2A; Supplementary Table S1 lists the GMT and 95% CI raw data used for this Figure). The neutralisation efficiency against the WT of the antibodies collected from HCW who had received three vaccine doses was significantly higher than that of infected unvaccinated HCW (Figure 2A and B). Following breakthrough infection of vaccinated individuals, the neutralisation efficiency, particularly against the WT virus, but also against the various Omicron variants, was further elevated (Figure 2B). The baseline neutralisation efficiency in HCW who had been immunised four times was significantly higher than that of HCW vaccinated three times (Figure 2B and C). Following infection, the neutralisation capacity against the WT virus and the various Omicron variants was significantly elevated (Figure 2C).

SARS-CoV-2: severe acute respiratory syndrome coronavirus 2.

The numbers over the bars indicate geometric mean titres. The whiskers indicate 95% confidence intervals.

**Figure 3:**
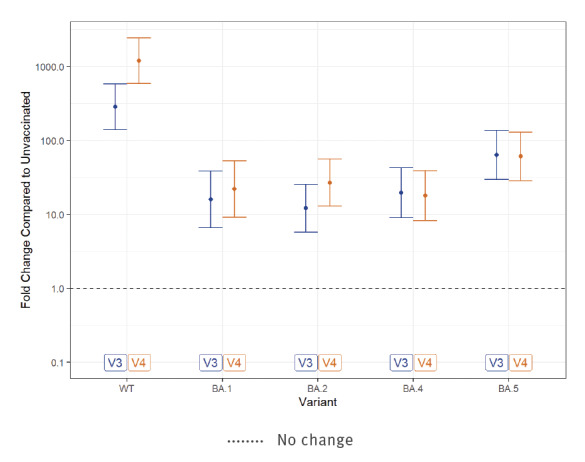
The fold change of SARS-CoV-2 neutralising antibody titres in sera from healthcare workers after COVID-19 vaccination and BA.1 infection, Israel, March–June 2022 (n = 41)

COVID-19: coronavirus disease; SARS-CoV-2: severe acute respiratory syndrome coronavirus 2.

Neutralisation efficiency against BA.5 was lowest among all Omicron variants, even among HCW infected with BA.1 following three or four vaccine doses (respectively GMT = 161; 95% CI: 104–229, Figure 2B and GMT = 154; 95% CI: 105–247, Figure 2C). Neutralisation efficiency against the WT virus was greater after four than three vaccinations (Figure 3). In contrast, neutralisation efficiency against BA.4 and BA.5 was similar in HCW vaccinated three or four times (Figure 3).

The fold-change in neutralising antibody titres of BA.1 convalescent HCW who previously received three or four BNT162b2 doses, compared with unvaccinated BA.1-convalescent HCW. The dashed line at 1 indicates no change.

## Discussion

The SARS-CoV-2 Omicron variant was discovered on 24 November 2021, in South Africa and declared a VOC by the World Health Organization. In the subsequent months, multiple lineages of the Omicron variants (BA.1, BA.2, BA.4 and BA.5) emerged. Omicron variants are highly transmissible and escapes neutralising antibodies induced by the currently available vaccines and previous SARS-CoV-2 infections [9].

Here, we evaluated neutralising antibody titres against SARS-CoV-2 WT and the four Omicron variants in unvaccinated individuals recovered from BA.1 and compared them with those of recovered individuals who had received three or four doses of vaccine before infection. Neutralisation efficiency against all Omicron variants was significantly higher in individuals who were vaccinated before BA.1 infection compared with unvaccinated recovered individuals. Neutralisation efficiency against BA.5 was lowest in all HCW and neutralisation efficiency against the WT was higher than against the Omicron variants. While it is not surprising that neutralisation efficiency against the WT virus increased with the number of vaccinations (as the spike RNA of the WT virus is present in the vaccine), neutralisation against Omicron variants was lower than against WT. This is probably because the spike protein of all Omicron variants is different than that of the WT virus [10]. Neutralisation efficiency was low against BA.2, BA.4 and BA.5 probably because of the D405N and F486V mutations.

It is important to note that the data for the third dose are from much longer after vaccination (5 months) than for the fourth dose (1 month), still Omicron neutralisation was more or less equal between the two groups. Still, some sera from people who had recovered from BA.1 infection did neutralise, and that in vitro neutralisation with serum only partially informs about the real protection against infection, as it does not inform about mucosal IgA, cellular responses or about protection against severe outcomes. 

## Conclusion

We report a low neutralisation efficiency against BA.4 and BA.5 even in sera obtained from BA.1-recovered HCW who previously received three or four vaccine doses. These findings suggest that an Omicron-specific vaccination might be indicated.

## Supplementary Data

132000 Supplement

## References

[1] Mor O Mandelboim M Fleishon S Bucris E Bar-Ilan D Linial M The rise and fall of a local SARS-CoV-2 variant with the spike protein mutation L452R. Vaccines (Basel). 2021;9(8):937. 10.3390/vaccines9080937 34452062 PMC8402656

[2] Tegally H Moir M Everatt J Continued emergence and evolution of Omicron in South Africa: New BA.4 and BA.5 lineages. medRxiv 2022 10.1101/2022.05.01.22274406

[3] Ou J Lan W Wu X Zhao T Duan B Yang P Tracking SARS-CoV-2 Omicron diverse spike gene mutations identifies multiple inter-variant recombination events. Signal Transduct Target Ther. 2022;7(1):138. 10.1038/s41392-022-00992-2 35474215 PMC9039610

[4] Tegally H Moir M Everatt J Giovanetti M Scheepers C Wilkinson E Emergence of SARS-CoV-2 Omicron lineages BA.4 and BA.5 in South Africa. Nat Med. 2022. 10.1038/s41591-022-01911-2 35760080 PMC9499863

[5] Van Goethem N Chung PYJ Meurisse M Vandromme M De Mot L Brondeel R Clinical severity of SARS-CoV-2 Omicron variant compared with Delta among hospitalized COVID-19 patients in Belgium during autumn and winter season 2021-2022. Viruses. 2022;14(6):1297. 10.3390/v14061297 35746768 PMC9227815

[6] Feikin DR Abu-Raddad LJ Andrews N Davies MA Higdon MM Orenstein WA Assessing vaccine effectiveness against severe COVID-19 disease caused by omicron variant. Report from a meeting of the World Health Organization. Vaccine. 2022 J;40(26):3516-27. 10.1016/j.vaccine.2022.04.069 35595662 PMC9058052

[7] Regev-Yochay G Gonen T Gilboa M Mandelboim M Indenbaum V Amit S Efficacy of a fourth dose of Covid-19 mRNA vaccine against Omicron. N Engl J Med. 2022;386(14):1377-80. 10.1056/NEJMc2202542 35297591 PMC9006792

[8] Cao Y Yisimayi A Jian F Song W Xiao T Wang L BA.2.12.1, BA.4 and BA.5 escape antibodies elicited by Omicron infection. Nature. 2022. 10.1038/s41586-022-04980-y 35714668 PMC9385493

[9] Yao L Zhu K-L Jiang X-L Wang X-J Zhan B-D Gao H-X Omicron subvariants escape antibodies elicited by vaccination and BA.2.2 infection. Lancet Infect Dis. 2022;22(8):1116-7. 10.1016/S1473-3099(22)00410-8 35738299 PMC9212811

[10] Fan Y Li X Zhang L Wan S Zhang L Zhou F . SARS-CoV-2 Omicron variant: recent progress and future perspectives. Signal Transduct Target Ther. 2022;7(1):141. 10.1038/s41392-022-00997-x 35484110 PMC9047469

