# Supplementary material for: COVID-19 vaccination and BA.1 breakthrough infection induce neutralising antibodies which are less efficient against BA.4 and BA.5 Omicron variants, Israel, March to June 2022
Source: Euro Surveill. 2022 Jul 28;27(30):2200559. doi: 10.2807/1560-7917.ES.2022.27.30.2200559 (PMC9336169; doi:10.2807/1560-7917.ES.2022.27.30.2200559)
Supplement: Supplement [file 22-00559_MANDELBOIM_Supplement.pdf]

Supplementary Appendix

This supplementary material is hosted by *Eurosurveillance* as supporting information alongside the article "Vaccination and BA.1 breakthrough infection induces neutralizing antibodies which are less efficient against BA.4 and BA.5 Omicron variants"on behalf of the authors who remain responsible for the accuracy and appropriateness of the content. The same standards for ethics, copyright, attributions and permissions as for the article apply. Supplements are not edited by *Eurosurveillance* and the journal is not responsible for the maintenance of any links or email addresses provided therein.

Supplementary Table 1: GMT and CI related to Figure 1

		Unvaccinated	3rd dose before infection	3rd dose after infection	4th dose before infection	4th dose after infection
Microneutraliztion assay WT	Geometrical mean	5.657	353.8	1625	2964	6810
	Upper and lower 95% CI	2.271-14.09	182.3-686.3	1118-2364	1663-5283	4709-9848
Microneutraliztion assay Omicron BA.1	Geometrical mean	42.22	12.13	147	168.9	933.6
	Upper and lower 95% CI	18.06-98.71	5.217-28.19	112.40-367.4	96.65-295.1	523.8-1664
Microneutraliztion assay Omicron BA.2	Geometrical mean	84.45	88.13	1024	436.3	2278
	Upper and lower 95% CI	55.59=128.3	51.86-149.8	623.1-1683	260.1-732	1206-4305
Microneutraliztion assay Omicron BA.4	Geometrical mean	8.980	10.68	176.9	74.25	161.3
	Upper and lower 95% CI	3.734-21.59	4.766-23.93	120.9-258.7	40.28-136.9	100.4-259.0
Microneutraliztion assay Omicron BA.5	Geometrical mean	2.520	3.560	161.3	49.97	154.0
	Upper and lower 95% CI	1.037-6.121	1.256-10.11	105.2-247.2	21.27-117.4	103.6-228.9
